# Lactate Is a Metabolic Mediator That Shapes Immune Cell Fate and Function

**DOI:** 10.3389/fphys.2021.688485

**Published:** 2021-10-18

**Authors:** Heather L. Caslin, Daniel Abebayehu, Julia A. Pinette, John J. Ryan

**Affiliations:** ^1^Department of Molecular Physiology and Biophysics, Vanderbilt University, Nashville, TN, United States; ^2^Department of Biology, Virginia Commonwealth University, Richmond, VA, United States; ^3^Department of Biomedical Engineering, University of Virginia, Charlottesville, VA, United States

**Keywords:** lactic acid, lactate, immunosuppression, Th17, immunometabolism, M2, immune, inflammation

## Abstract

Lactate and the associated H^+^ ions are still introduced in many biochemistry and general biology textbooks and courses as a metabolic by-product within fast or oxygen-independent glycolysis. However, the role of lactate as a fuel source has been well-appreciated in the field of physiology, and the role of lactate as a metabolic feedback regulator and distinct signaling molecule is beginning to gain traction in the field of immunology. We now know that while lactate and the associated H^+^ ions are generally immunosuppressive negative regulators, there are cell, receptor, mediator, and microenvironment-specific effects that augment T helper (Th)17, macrophage (M)2, tumor-associated macrophage, and neutrophil functions. Moreover, we are beginning to uncover how lactate and H^+^ utilize different transporters and signaling cascades in various immune cell types. These immunomodulatory effects may have a substantial impact in cancer, sepsis, autoimmunity, wound healing, and other immunomodulatory conditions with elevated lactate levels. In this article, we summarize the known effects of lactate and H^+^ on immune cells to hypothesize potential explanations for the divergent inflammatory vs. anti-inflammatory effects.

## Introduction

The field of physiology has long recognized the importance of metabolic pathways for energy sustaining adenosine triphosphate (ATP) production in homeostasis and in response to physiological stressors. However, the importance of cell metabolism within immunology has only become more appreciated in the past decade (O’Neill et al., 2016). We now understand that immune cells differentially utilize glycolysis vs. oxidative phosphorylation (OX PHOS) for differentiation, polarization, and effector functions (Norata et al., 2015; Loftus and Finlay, 2016). Moreover, we are just beginning to understand the preferential use of different substrates and the full functionality of different metabolites.

Lactate, once considered a metabolic waste product, is not only produced during glycolytic ATP production, but can be used for energy production, gluconeogenesis, and autocrine, paracrine, and endocrine signaling (Gladden, 2004; Brooks, 2018). In 1985, the cell-to-cell lactate shuttle theory introduced the idea that lactate can be produced in one [muscle] cell type and consumed in another (Brooks, 1985, 2009). We now know this to be true in the immune system as well. Lactate is elevated in inflammatory diseases due to increased production or impaired clearance, which then influences immune cell function. Several recent reviews have discussed the role of lactate in immune cell activation within one specific disease context (Pucino et al., 2017; Brooks, 2018; Santos et al., 2019; Baltazar et al., 2020; Ivashkiv, 2020). Thus in the current work, we have reviewed multiple cell types and disease models to help shape our overall understanding of how lactate influences immune cell function. Importantly, we offer potential explanations for the seemingly contradictory pro- and anti-inflammatory functions of lactate and lactic acid.

While lactate, nicotinamide adenine dinucleotide (NAD+), and ATP are considered primary metabolic products of glycolytic metabolism, there is also a concurrent release of H^+^ ions (Kemp, 2005; Kemp et al., 2006; Brooks, 2018; Qian, 2018). From an immunological perspective, both lactate and H^+^ ions appear important for cellular function and feedback, as they can act separately or together to influence immune function. Thus, this review will cover immunological studies of lactate alone and lactate with the associated H^+^ ions. Although lactic acid is always dissociated at physiological pH, this terminology will be specifically used when lactic acid was added to culture systems or animal models with the understanding that dissociation occurs and results are due to both ions (see specific experimental details described in Supplementary Table 1 and below).

## Receptor Transport and Metabolism

There are multiple mechanisms by which lactate and the associated H^+^ ions can enter immune cells. Proton-dependent monocarboxylate transporters (MCTs) are the primary proteins known to facilitate H^+^-dependent transport of monocarboxylates, such as lactate, down their concentration gradients (Halestrap, 2012, 2013; Sun et al., 2017). MCT-1 (Slc15a) is the primary lactate importer, and MCT-4 (Slc16a3) is the primary lactate exporter, both with ubiquitous cell expression (Halestrap, 2012; Contreras-Baeza et al., 2019). MCT-1 has been shown to mediate the effects of lactate and the associated H^+^ ions in macrophages, mast cells, and CD8^+^ T cells (Colegio et al., 2014; Haas et al., 2015; Abebayehu et al., 2016; Caslin et al., 2019; Zhang et al., 2019b).

In addition to transport *via* MCT-1, lactate can also be transported through sodium-dependent transporters (Slc5a12) on CD4^+^ T cells (Haas et al., 2015), and can activate G-protein coupled receptor (GPR)81 on monocytes, macrophages, and dendritic cells (Hoque et al., 2014; Brown et al., 2020). Moreover, tissue acidification by H^+^ ions is sensed by GPR65 and GPR132 on macrophages (Chen et al., 2017; Bohn et al., 2018). These receptors transport different substrates and have selective expression on immune cells (Haas et al., 2015), which has been proposed to orchestrate differential functional responses by different immune cell types (Pucino et al., 2017).

**Figure 1:**
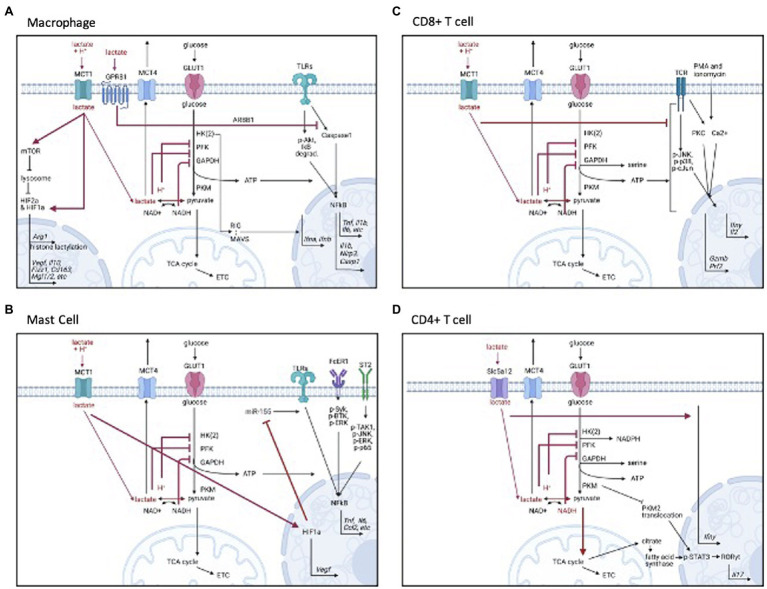
Signaling mechanisms of lactate by cell type. **(A)** Monocytes and macrophages, **(B)** Mast cells, **(C)** CD8+ T cells, and **(D)** CD4+ T cells. Figure created with BioRender.com.

Once inside the cell, a buildup of lactate and the associated H^+^ ions generally act as negative feedback regulators for glycolytic ATP production. H^+^ ions inhibit phosphofructokinase activity (Dobson et al., 1986; Leite et al., 2011), and lactate is converted to pyruvate by lactate dehydrogenase, which impairs NADH recycling (Gray et al., 2014; Angelin et al., 2017). Because NAD^+^ is necessary for glyceraldehyde 3-phosphate dehydrogenase (GAPDH) function, NAD^+^ is needed for sustained glycolysis (Quinn et al., 2020). Lactate and the associated H^+^ ions have similar inhibitory effects on glycolysis in immune cells (Figure 1). Lactic acid, which rapidly dissociates to lactate and H^+^, suppressed glycolysis in the myeloid lineage and T cells, as measured by Seahorse metabolic flux analysis (Dietl et al., 2010; Errea et al., 2016; Ratter et al., 2018; Fischbeck et al., 2020; Quinn et al., 2020). Lactic acid suppressed glucose uptake, lactate export, glycolytic enzyme expression, and intracellular ATP levels in monocytes and mast cells (Dietl et al., 2010; Caslin et al., 2019). Further, exogenous lactate reduced the NAD^+^/NADH ratio in activated CD4^+^ T cells, suggesting NADH-to-NAD^+^ recycling was inhibited, leaving less available NAD^+^ for the continuation of glycolysis (Pucino et al., 2019). Impaired NADH recycling also limited serine production, which was important for T cell activation (Quinn et al., 2020).

In the past 10–15years, immunologists have begun to fully appreciate how bioenergetic pathways are linked with inflammatory function. Generally, glycolysis fuels inflammatory cells and oxidative phosphorylation supports anti-inflammatory, regulatory cells (O’Neill et al., 2016). This suggests that lactic acid-mediated glycolytic inhibition may suppress inflammatory immune cell function and promote regulatory functions.

## Immune Effects and Signaling Mechanisms

**Figure 2:**
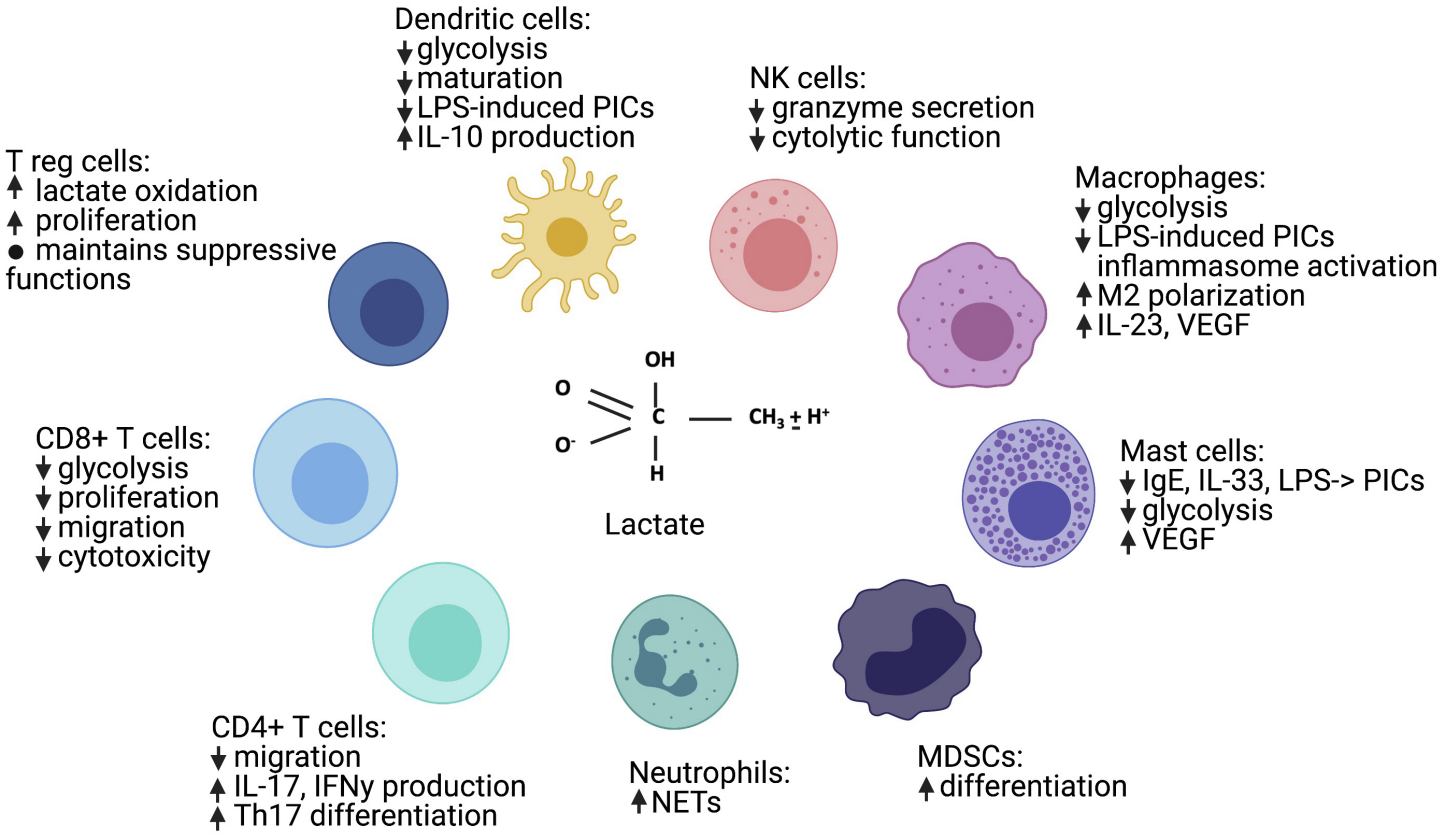
Lactate has many immune cell-specific effects, ranging from general immunosuppression to Th17 and M2 polarization. “↑” indicates a positive signal or induction, “↓” indicates a negative signal or inhibition, and “PICs” indicates “pro-inflammatory cytokines.” Figure created with BioRender.com.

Many publications support the idea that lactic acid and lactate are generally immunosuppressive. However, more recent publications suggest that cell, receptor, and microenvironmental effects also determine how lactate and lactic acid influence inflammatory macrophage, neutrophil, and T helper (Th)17 cell function. Lactic acid not only inhibits glycolytic energy production, but additional mechanisms of action occur *via* changes in signaling cascades and epigenetic modifications. In this section, we will summarize the literature by cell type, describe the mechanisms of action, and discuss potential explanations for seemingly opposing findings. Please see Figure 2 for a general summary of the effects of lactate and lactic acid on immune cell subsets, Supplementary Table 1 for specific study details and findings, and Figure 1 for signaling cascades that contribute to the effects of lactic acid on immune function in specific cell types.

### Myeloid Immune Cells

#### Monocytes and Macrophages

The effects of lactic acid and lactate have been most studied on innate myeloid cells. In monocytes and macrophages, lactic acid suppressed an array of lipopolysaccharide (LPS)-induced cytokine and chemokine mediators (Dietl et al., 2010; Peter et al., 2015; Errea et al., 2016). Lactate itself also suppressed inflammasome assembly, LPS-stimulated cytokine secretion, and migration in macrophages and monocytes (Goetze, 2011; Hoque et al., 2014; Ratter et al., 2018). Interestingly, Dietl et al. (2010) found the effects of lactic acid on human monocytes to be pH-dependent, which is supported by two studies on acidity in alveolar macrophages (Bidani, et al., 1998; Fernandez et al., 2013). Conversely, Peter et al. (2015) found lactic acid, lactate, and acidity to have differential effects depending on the gene of interest. Thus, both lactate and the associated H^+^ ion may influence macrophage function.

Lactic acid and lactate can suppress not only glycolysis, but specific receptor signaling cascades in macrophages and monocytes (see Figure 1A). The mechanisms of action are varied. For example, lactic acid inhibited LPS receptor signaling by delaying protein kinase B (AKT) phosphorylation, inhibitor of nuclear factor kappa B (IκB-alpha) degradation, and nuclear factor kappa B (NFκB) nuclear accumulation and activation (Hoque et al., 2014; Peter et al., 2015). Lactate has also impaired toll-like receptor (TLR)-4 mediated inflammasome assembly by eliciting GPR81-induced inhibitory signals (Hoque et al., 2014; Zhang et al., 2019b). Also, lactate can directly interact with the mitochondrial antiviral-signaling (MAVS) protein, preventing MAVS aggregation and therefore reducing type I interferon production during RIG-I-like receptor (RLR) signaling (Zhang et al., 2019b). Together these data show the variety of mechanisms by which lactate and the associated H^+^ ion can influence macrophage activation.

Lactic acid not only suppresses inflammatory macrophage (M1) function; it enhances regulatory, or anti-inflammatory, M2 polarization. Multiple publications have shown that lactic acid induced M2-associated genes (Colegio et al., 2014; Bohn et al., 2018; Zhang and Li, 2020). This polarization was dependent on MCT transport, hypoxia inducible factor (HIF) activation, and inducible cyclicAMP early repressor (ICER) induction (Colegio et al., 2014; Bohn et al., 2018; Liu et al., 2019; Zhang and Li, 2020). HIFs are transcription factors known to regulate both metabolic and inflammatory genes, and ICER is a transcriptional repressor that inhibits TLR-dependent NFκB signaling (Corcoran and O’Neill, 2016; Bohn et al., 2018). These data suggest that there are multiple regulatory control points for lactate within signaling cascades and gene transcription that contribute to observed effects. Additionally, arginase *(Arg)-1* transcription has been induced following histone lactylation, a lysine modification that occurs 16–24h following M1 activation (Zhang et al., 2019a). Thus, lactate and the associated H^+^ ions appear to be an intrinsic regulatory feedback pathway to help reduce macrophage inflammation and restore homeostasis.

In contrast to the above immunosuppressive effects, stimulatory effects of lactic acid and lactate on myeloid cell populations have also been reported. Lactic acid has been repeatedly shown to increase LPS-induced IL-23 production in monocytes, macrophages, and tumor infiltrating immune cells (Shime et al., 2008; Witkin et al., 2011; Peter et al., 2015). Moreover, lactate has been shown to increase prostaglandin E_2_ (PGE2) synthesis (Wei et al., 2015). Together, these data suggest a mediator-specific effect. However, lactate has also been shown to enhance the LPS-induced secretion of IL-6, matrix metalloproteinase (MMP)1, and IL-1β from U397 cell lines and human monocyte-derived macrophages (Nareika et al., 2005; Samuvel et al., 2009) and to increase NFκB activity *via* MCTs (Samuvel et al., 2009), which is in direct contrast to the above publications. It is not entirely clear why these publications found inflammatory effects of lactic acid, while others repeatedly reported anti-inflammatory effects. Both the Nareika and Samuvel publications out of the Huang group used U397 cell lines, which are derived from a histiocytic lymphoma, and thus may have intrinsic characteristics that differ from murine and human monocytes and macrophages (Nareika et al., 2005; Samuvel et al., 2009). Samuvel et al. (2009) also assessed human monocytes-derived macrophages, and it is possible that the observed effects may have been influenced by *ex vivo* differentiation or culture conditions—such as the inclusion of insulin and glutamine in culture media.

This hypothesis that the microenvironment may influence the effects of lactate is supported by two additional studies in the tumor immunology field. When monocytes were differentiated in the presence of lactate with granulocyte-macrophage colony-stimulating factor (GM-CSF), or adenocarcinoma-conditioned media (with elevated lactate and many other mediators), they increased both inflammatory (M1) and regulatory (M2) mediators, consistent with a tumor-associated macrophage (TAM) phenotype (Penny et al., 2016; Paolini et al., 2020). Further experimentation showed that GM-CSF and lactate together drove IL-6-dependent macrophage colony-stimulating factor (M-CSF) production and consumption, which promoted an inflammatory feed-forward loop. It is plausible that other factors in the tumor-conditioned media also had a similar impact. Thus, these two studies suggest that soluble mediators in the microenvironment may influence lactate effects, especially during differentiation.

#### Dendritic Cells and Myeloid-Derived Suppressor Cells

Similar to the inhibitory effects reported in monocytes and macrophages, exogenous and endogenous lactic acid reduced dendritic cell maturation and suppressed LPS-induced cytokine production (Gottfried, 2006; Nasi et al., 2013). Tumor-derived lactate also activated the receptor GPR81 on dendritic cells to reduce antigen presentation, cytokine production, and cyclic adenosine monophosphate (cAMP) activation (Brown et al., 2020). Additionally, tumor-derived lactic acid may also augment T helper (Th)2 polarization by dendritic cells (Selleri et al., 2016). This further supports the M2 polarization data discussed above and suggests that lactic acid may promote a general Th2/M2 regulatory response. Furthermore, lactate augmented differentiation of myeloid-derived suppressor cells (Husain et al., 2013), providing more support for a general inhibitory effect in myeloid cells.

#### Mast Cells

Recent publications from our laboratory have extended these findings to mast cells. We have shown that lactic acid suppresses immunoglobulin (Ig)E- and IL-33-dependent inflammatory cytokine and chemokine production (Abebayehu et al., 2016, 2019). Moreover, lactic acid suppressed inflammatory cytokine and chemokine production following TLR activation by bacterial and viral stimuli (Caslin et al., 2019). Interestingly, lactic acid also increased the secretion of the angiogenic factor vascular endothelial growth factor (VEGF) following IgE and IL-33 activation, supporting the reported macrophage M2 polarization data above. The suppressive effects of lactic acid in mast cells were dependent on MCT-1, and were reproduced by acidification or high concentrations of lactate (Abebayehu et al., 2016; Caslin et al., 2019). Additionally, inhibiting glycolysis mimicked lactic acid, while increasing ATP availability reversed lactic acid effects on LPS signaling (Caslin et al., 2019). These latter data suggest that suppressing glycolytic ATP production may be necessary and sufficient for lactic acid effects.

Our data also support and extend the signaling cascades and epigenetic mechanisms by which lactic acid can suppress myeloid activation (see Figure 1B). Similar to macrophages, we found that lactic acid reduced NFκB activity downstream of LPS (Caslin et al., 2019) and promoted HIF-1α-dependent VEGF production (Abebayehu et al., 2016, 2019). We also reported that lactic acid inhibits kinase activation downstream of the IL-33 and IgE receptors. In addition, lactic acid reduced microRNA (miR)-155-5p expression following IL-33 and LPS activation. miR-155 diminishes expression of inhibitory proteins (Huffaker and O’Connell, 2015; Lind et al., 2015), and thus amplifies inflammatory signaling. In our studies, a miR-155-5p mimic reversed the suppressive effects of lactic acid, indicating lactic acid can preserve negative feedback pathways partly by suppressing miR-155-5p. Interestingly, lactic acid still inhibited LPS-induced cytokine production in miR-155 knockout mice, suggesting that lactic acid may act by multiple redundant mechanisms.

In another recent publication, lactic acid suppressed calcium mobilization, degranulation, and the release of chemokines and cytokines through the MAS-related GPR family member X2 (MRGPRX2) receptor in culture (Syed et al., 2021). Lactic acid also reduced passive systemic anaphylaxis to compound 48/80 and skin inflammation in a mouse model of rosacea. Together with our results, these data suggest that lactic acid suppresses mast cell-mediated inflammation.

#### Neutrophils

The effects of lactate have also been studied in neutrophils. Interestingly, endogenous and exogenous lactate treatment induced neutrophil extracellular trap (NET) formation (Awasthi et al., 2019), an inflammatory function aiding bacterial and viral clearance. The mechanisms by which lactate induced NET formation are not known. As glycolysis is needed for NET production (Rodríguez-Espinosa et al., 2015), these results may seem surprising. However, the formation of superoxide as a reactive oxygen species can also induce NET formation (Al-Khafaji et al., 2016), and glycolytic inhibition promotes oxidative stress in many cell types (Le et al., 2010; Korga et al., 2019). The relative contribution of glycolysis and oxidative stress to NET formation and lactate action may be an interesting area of study for the future research. Importantly, these findings further suggest that lactate effects are cell-specific and can be pro-inflammatory.

### Lymphoid Immune Cells

#### Natural Killer Cells and Cytotoxic (CD8^+^) T Cells

In the lymphoid compartment, the effects of lactic acid and lactate have been studied in NK and T cells. In NK cells, lactic acid (both lactate and acidity) inhibited cytolytic function (Husain et al., 2013). In CD8^+^ T cells, lactic acid reduced proliferation, degranulation, motility, cytolytic activity, and inflammatory mediator secretion (interferon (IFN)γ, perforin, and granzyme; Fischer et al., 2007; Mendler et al., 2012; Haas et al., 2015; Fischbeck et al., 2020). In CD8^+^ T cells, these effects are likely due to altered receptor signaling, as lactic acid suppressed protein kinase activity (Mendler et al., 2012), and suppression was dependent on MCT-1 (Haas et al., 2015; for signaling, see Figure 1C). Interestingly, glycolytic suppression was not shown to be required for all lactic acid effects in CD8^+^ T cells (Haas et al., 2015), suggesting that other mechanisms of action beyond metabolism—such as cell signaling—should be explored.

#### Helper (CD4^+^) T Cells: T Regulatory and T Helper 17 Cells

In the CD4^+^ T cell lineage, lactic acid and lactate have differential effects. Lactate suppressed CD4^+^ cell motility while increasing IL-2 secretion (Roth and Droge, 1991; Haas et al., 2015), which can promote T cell differentiation, including that of T regulatory cells (Treg; Chinen et al., 2016). Tregs have also been shown to uptake and metabolize lactate to maintain suppressor function in high lactate conditions, like the tumor microenvironment (Watson et al., 2021). Specifically, Tregs converted lactate into pyruvate, citrate, and malate to fuel the tricarboxylic acid (TCA) cycle and into phosphoenolpyruvate for glycolytic intermediates essential for proliferation. This could logically extend the findings of lactic acid-mediated suppression of NK and CD8^+^ T cells, because Tregs also inhibit NK- and CD8^+^ T cell activity (Plitas and Rudensky, 2016).

Despite promoting Treg-mediated inhibition, lactate can promote the differentiation of naive CD4^+^ T cells into inflammatory Th17 cells (for signaling, see Figure 1D). Two recent studies suggest that sodium lactate increased *Il17* and *Ifn*γ gene expression *via* retinoic acid receptor-related orphan receptor gamma (RORγT), suggesting Th17 polarization (Haas et al., 2015; Pucino et al., 2019). Unlike CD8^+^ cells, which express MCT-1, CD4^+^ T cells express Scl5a12, a sodium-coupled lactate transporter, which may explain the opposite effects of lactic acid and lactate on inflammatory function in these cell types (Haas et al., 2015). Through Slc5a12, lactate not only blunted glycolytic energy production, but increased oxidative stress, promoting the translocation of pyruvate kinase into the nucleus to phosphorylate signal transducer and activator of transcription (STAT3/1), and inducing RORγt-dependent IL-17 transcription (Pucino et al., 2019). Interestingly, lactate-induced IL-17 production was also due to fatty acid synthesis *via* the pentose phosphate pathway, nicotinamide adenine dinucleotide phosphate (NADPH), and TCA-derived citrate, suggesting the involvement and coordination of many metabolic pathways. These studies add considerable detail to understanding how lactate interacts with metabolism to alter cellular function.

It is not yet clear how lactate controls both Treg suppressor functions and inflammatory Th17 differentiation; however, there is a unique relationship between both cell types (reviewed in DeBerardinis et al., 2007). Treg and Th17 differentiation both require TGFβ, which can induce T cells expressing the lineage-defining transcription factors for both Treg (Foxp3) and (RORγt). Cytokines in the microenvironment drive further differentiation, with IL-2 inducing Tregs and IL-6 and IL-21 inducing Th17 cells. Moreover, Tregs and Th17 can trans-differentiate in some situations. Different utilization of bioenergetic pathways, like the hexosamine pathway, and different metabolite-induced epigenetic landscapes appear to contribute to the development of Treg vs. Th17 cells (DeBerardinis et al., 2007). However, the role of lactate is poorly understood. Future studies should consider how lactate promotes the development of each cell type and the role of the microenvironment in shaping that response.

### Discussion of Cell-Specific Effects

The opposing pro- and anti-inflammatory effects of lactate with and without the associated H^+^ ions have several possible explanations. For example, the divergent effects on CD8^+^ and CD4^+^ T cells appear due to selective MCT-1 vs. Scl5a12 expression, respectively (Haas et al., 2015). It is possible that transporter diversity also explains other divergent findings. Lactic acid suppression was MCT-1 dependent in many myeloid populations; however, receptor dependency was not measured in all studies. It would be particularly interesting to know which receptors are required for myeloid cell IL-23 induction, the mixed tumor-associated macrophage phenotype, and enhanced neutrophil NET formation.

In addition to transporter expression, acidity has been linked to differential effects. Some studies reported that lactic acid effects are pH-dependent (Dietl et al., 2010), while others have found the effects of lactic acid, lactate alone, and acidity specific to the gene of interest (Peter et al., 2015). We hypothesize that some of these differential effects are due to substrate concentrations and receptor kinetics. The predominant role of MCT transporters is proton-linked transport of L-lactate, but at higher concentrations, MCT can exchange one monocarboxylate molecule for another without net proton movement (Halestrap, 2012). In our studies, 12.5-mM lactic acid suppressed cytokine production and reduced the pH of the buffered media to 6.7 (Caslin et al., 2019). It remained significantly lower than control media for 1h. Fischbeck et al. (2020) observed a similar drop in pH with 10-mM lactic acid and a drop to pH 6.3 with 20-mM lactic acid addition. Additionally, we observed that formic acid, with a similar pKa, also suppressed IL-6 secretion, and sodium lactate above 20mM could mimic lactic acid. The roles of acidity and concentration are supported in dendritic cells, where the inhibitory effect of 10-mM lactic acid was reversed by adjusting the pH to 7.4, but adjusting the pH had less effect at concentrations above 10mM (Gottfried, 2006). Moreover, sodium lactate suppressed LPS-induced cytokine production, but only when present for a prolonged period of time (Ratter et al., 2018), whereas we found that lactic acid could suppress cytokine production when added simultaneously, or even after activation (Caslin et al., 2019). These data suggest that in addition to differential transporter expression, concentration and acidity can alter the outcome using the same transporter. Lactate and the associated H^+^ ions can act at lower concentrations, while lactate alone can mimic these effects when present at higher concentrations. Future studies should more thoroughly investigate the effects of lactate vs. pH as well as the receptor utilized by each cell type.

Another set of seemingly contradictory results is the influence of lactate with and without the associated H^+^ ions on HIF-1α and HIF-2α. These transcription factors are commonly upregulated in hypoxic environments to enhance glucose and iron metabolism, angiogenesis, and erythropoiesis. In myeloid cells, HIF-1α generally promotes glycolysis, increases pro-inflammatory gene expression, and mediates bacterial killing (Imtiyaz and Simon, 2010). Distinct from these hypoxia-induced effects, our laboratory and others have shown that lactic acid induces HIF-1α function while suppressing glycolysis and inflammatory cytokine production (Colegio et al., 2014; Abebayehu et al., 2016, 2019). The selective induction of HIF-1α-dependent VEGF production suggests specific transcriptional effects. Furthermore, HIF-1α has been shown to promote Th17 polarization (Shi et al., 2011; Corcoran and O’Neill, 2016). Thus, a lactate-HIF connection appears to control multiple axes of inflammation and angiogenesis. HIFs are controlled at multiple levels, with both transcriptional and post-translational regulation, with additional environmental factors like iron availability influencing HIF degradation (Siegert et al., 2015). Thus, lactate and associated H^+^ ions may access multiple levels of HIF regulation and could offer insight into how these important transcription factors control inflammation and angiogenesis.

Finally, the biological rationale for promoting Th17, Th2, and M2 responses is unclear and likely represents an opportunity to better understand fundamental aspects of immunity. Lactic acid induces both Th17 differentiation and myeloid IL-23 production (which promotes Th17 differentiation; Shime et al., 2008; Haas et al., 2015; Pucino et al., 2019). In apparent contrast to this, lactic acid not only induces M2 polarization (Colegio et al., 2014; Bohn et al., 2018; Zhang and Li, 2020), but also augments dendritic cell-mediated Th2 polarization (Selleri et al., 2016). Th17 and Th2/M2 responses are often seen as acting in opposition. However, recent studies suggest that Th17 responses are crucial for intestinal hypercontractility and worm expulsion in anti-helminth immunity (Allen et al., 2015; Steel et al., 2019), which traditionally requires Th2 responses. Moreover, IL-17- and Th2-type cytokines can enhance or counter-regulate each other in the response to helminths (Allen et al., 2015). Adding another layer to this model system, lactic acid also suppresses mast cell IgE- and IL-33-induced responses (Abebayehu et al., 2016, 2019), which contribute to anti-helminth immunity. Induction of both Th17 and Th2/M2, in addition to suppressing mast cells, suggests either cooperativity between these branches of immunity, an attempt to maintain homeostasis, or perhaps both at different periods of the infection. Future studies should examine the contribution of cell, receptor, mediator, and microenvironment-specific effects, and should aim to uncover a more complete understanding of the biological role of lactic acid on the different types of immunity.

## Implications for Disease

Lactate is elevated in many disease states, either systemically or locally, *via* enhanced production and/or impaired clearance. Lactate is produced and consumed by many different cell types, and the effects of lactate on a variety of cell types likely play a role in disease pathogenesis and prognosis. We will primarily focus here on the immune-specific effects of lactate and lactic acid, with an effort to differentiate lactate and pH effects. Some studies have injected lactic acid into animal models, and H+ ions are likely produced alongside lactate in many animal models. The pH effects can be buffered by bicarbonate or other molecules, but this varies with the organ involved. We hypothesize that lactate is a negative feedback regulator in acute inflammatory conditions. However, it is important to remember that by definition, chronic disease states represent a loss of homeostasis, making it difficult to untangle cause and consequence from association.

### Cancer

In the 1920s, it was initially shown that tumor cells consumed substantial glucose and secreted lactate even in the presence of adequate oxygen (Cori and Cori, 1925; Warburg et al., 1927). This is now known as the Warburg effect (Potter et al., 2016), and is a hallmark characteristic of cancer (Hanahan and Weinberg, 2011). By making ATP from glycolysis, cancer cells can use metabolic intermediates from other pathways for proliferation (Potter et al., 2016). In the tumor microenvironment, lactate levels can reach 40mM (Ostroukhova et al., 2012), with extracellular pH as low as 6–6.5 (Wike-Hooley et al., 1984). This is a striking comparison to normal cell environments with a lactate level~1mM and pH of 7.2. Additionally, high lactate levels are associated with increased metastasis and decreased survival (Walenta et al., 2000, 2004), suggesting that lactate may be used as a clinical prognostic parameter.

Lactate can not only provide a survival advantage for tumor cells by upregulating oncogenes and inducing angiogenesis; it promotes immune evasion and is considered an oncometabolite (Choi et al., 2013; San-Millán and Brooks, 2017; San-Millán et al., 2019). Lactic acid from the tumor microenvironment suppressed CD8^+^T cell activation and tumor killing (Fischbeck et al., 2020). Additionally, lactic acid promoted both M2 differentiation and a mixed M1/M2 macrophage phenotype characteristic of tumor-associated macrophages that aid in immune escape (Colegio et al., 2014; Helm et al., 2014; Penny et al., 2016; Mu et al., 2018; de-Brito et al., 2020; Paolini et al., 2020). Lactate and lactic acid also augmented HIF-1α-mediated VEGF production from mast cells (Abebayehu et al., 2016, 2019) and macrophages (Colegio et al., 2014) which may contribute to angiogenesis and tumor growth. Moreover, lactate enhanced the synthesis of prostaglandin E2 (PGE2) by cyclooxygenase (COX)2 in monocytes, which is involved in tumor progression and the development of therapeutic resistance (Wei et al., 2015; Tong et al., 2018). Together, these immunosuppressive effects select for tumor growth and escape, migration, invasion, and immune evasion (Choi et al., 2013; Biswas, 2015; Mu et al., 2018). Interestingly, many known chemotherapeutic agents are weak bases, whose ionization in the acidic tumor environment reduces uptake and efficacy (Raghunand et al., 1999; Sun et al., 2017).

There have been a few approaches *in vivo* to directly target lactic acid effects on tumor growth and immune function. Bicarbonate added to drinking water has been shown to reduce melanoma tumor size, increase tumor-associated CD8^+^ cells, and enhance survival in mice (Pilon-Thomas et al., 2016). Combining bicarbonate therapy with immunotherapy for melanoma or doxorubicin treatment for breast cancer appears to augment drug effects (Raghunand et al., 1999; Pilon-Thomas et al., 2016). Furthermore, proton pump inhibitors, which can increase tumor pH, significantly increased survival and T cell function in a murine melanoma model (Calcinotto et al., 2012). Finally, diclofenac, a lactate dehydrogenase (LDH)A inhibitor, has been used in a murine glioma model to reduce lactic acid secretion, effectively enhancing DC inflammatory capacity and reducing the accumulation of Tregs (Chirasani et al., 2013). However, the therapy also suppressed T cell glycolysis, compromising IFN-γ production and T-cell proliferation. This highlights the importance of glycolysis for immune cell function and suggests therapeutics should specifically target lactate consumption or signaling to enhance immune function, instead of targeting lactate production. Preventing lactate actions on T cells and macrophages without suppressing glycolysis should enhance anti-tumor immunity.

### Wound Healing

Lactate levels are also elevated in the local wound environment, typically reported around 20mM, with a range between 5 and 80 mM (Löffler et al., 2011; Britland et al., 2012). This is due to poor tissue perfusion, poor oxygenation, or atypical bacterial colonization and immune activation (Britland et al., 2012). As stated, lactate induced M2 polarization and VEGF production (Constant et al., 2000; Trabold et al., 2003; Hunt et al., 2007; Porporato et al., 2012; Colegio et al., 2014; Abebayehu et al., 2016, 2019). In contrast to cancer, these effects are beneficial and promote angiogenesis, endothelial cell migration, and wound closure (Hunt et al., 2007; Porporato et al., 2012). Additionally, lactate enhanced fibroblast proliferation, myofibroblast differentiation, and collagen deposition (Trabold et al., 2003; Wagner et al., 2004; Kottmann et al., 2012), which also contribute to wound healing.

Local and systemic lactate delivery *via* lactate-releasing polymers can promote angiogenesis, endothelial progenitor cell recruitment, procollagen activation, and extracellular matrix deposition in mice with ischemic wounds (Trabold et al., 2003; Porporato et al., 2012). Interestingly, poly(lactic-co-glycolic acid; PLGA) nanoparticle delivery of VEGF can accelerate non-diabetic and diabetic wound healing faster than either VEGF or PLGA lactate-based polymers alone (Chereddy et al., 2015). While these therapeutics are promising, further understanding of the mechanisms by which lactate and the associated H^+^ ions can induce immune resolution and repair may help develop even better wound treatments.

### Sepsis

Sepsis is a pathological inflammatory response to systemic infection. One hallmark is elevated lactate levels due to tissue hypoperfusion, impaired pyruvate dehydrogenase activity, elevated catecholamine secretion, and increased immune cell activation (Vary, 1996; Haji-Michael et al., 1999; McCarter et al., 2001). Blood lactate concentrations in sepsis are often between 2 and 10 mM; however, concentrations have been reported as high as 20mM due to the timing of measurement and severity of disease (Filho et al., 2016; Kuttab et al., 2018; Theerawit et al., 2018). Many studies show that elevated blood lactate (≥4mM) and impaired clearance are independently associated with increased mortality in septic patients (Nguyen et al., 2004; Trzeciak et al., 2007; Arnold et al., 2009; Nichol et al., 2011; Marty et al., 2013). Thus, the use of lactate clearance as a treatment guideline for sepsis has gained traction in adult and pediatric patients alike (Rhodes et al., 2017; Kuttab et al., 2018; Nazir et al., 2019). It is not fully understood if lactate is a cause or a consequence of sepsis. As articulated by Brooks, hyperlactemia is often a strain on the system to lessen the effects of injury. However, while this may be protective during the initial cytokine storm, it can be pathological in the late stage of sepsis (Brooks, 2018; Caslin et al., 2019).

We and others reported that lactic acid and lactate suppressed LPS-induced cell metabolism and immune cell function, which may impair antibacterial defense mechanisms (Gottfried, 2006; Peter et al., 2015; Errea et al., 2016; Caslin et al., 2019). We have also shown that intraperitoneal lactic acid administration prior to LPS injection suppressed cytokine production in mice (Caslin et al., 2019). Similarly, sodium lactate suppressed cytokine production in a rat model of sepsis (Besnier et al., 2020). Intratracheal acidic aspiration has also been shown to impair clearance of *S. pneumoniae* and *E. coli* (Fernandez et al., 2013). Together with the clinical observations above, these data support the theory that high lactate levels early in sepsis may act to suppress immune cell glycolysis and function. However, this negative regulation is detrimental, impairing pathogen clearance, and contributing to immunosuppression observed in the secondary phase of sepsis. This latter phase is marked by reduced glucose metabolism, cytokine production, antigen presentation, and cytolytic function (Hotchkiss et al., 2013a,b), and thus resembles the effects of lactate and lactic acid treatment. Separate from this general suppressive activity, the ability of lactate or lactic acid to augment NET formation and IL-17 production, while impairing CD8^+^ degranulation and mobilization (Haas et al., 2015; Awasthi et al., 2019; Pucino et al., 2019), may also explain how the septic cytokine storm can be disconnected from bacterial clearance. Future studies should directly examine the effects of early lactate clearance on *ex vivo* immune cell metabolism and function in septic patients during the immunosuppressive phase of the disease. Additionally, future studies should attempt to reconcile these potentially detrimental immune effects with the seemingly beneficial effects of lactate metabolism on other organ systems (Garcia-Alvarez et al., 2014; Hernandez et al., 2019; See and Bellomo, 2021). Lactate may be increased *via* oxidation and gluconeogenesis to maintain organ function. Thus, future therapeutics could not only attempt to reduce lactate uptake or signaling in immune cells, but aim to selectively increase oxidation in non-immune cells.

### Ulcerative Colitis and Rheumatoid Arthritis

Lactate levels are elevated in many autoimmune diseases, including ulcerative colitis and rheumatoid arthritis. In patients with ulcerative colitis, those with moderate and severe colitis have low fecal pH and high fecal lactate, produced by inflamed colonic mucosal cells (Vernia et al., 1988; Hove et al., 1995). In an experimental mouse model of colitis, knocking out the lactate receptor GPR81 increased inflammatory cytokine production in intestinal dendritic cells and macrophages and worsened colonic inflammation (Ranganathan et al., 2018). Pharmacological activation of GPR81 reduced colonic inflammation. Similarly, lactate administration prior to murine colitis onset reduced inflammation and serum IL-6 (Iraporda et al., 2016). Together, these data suggest that lactate may act as a negative feedback regulator limiting colitis inflammation.

Arthritic joints have also long been recognized as a site of high lactate (Goetzi et al., 1971; Treuhaft and McCarty, 1971), due to rapid synovial fibroblast turnover and proliferation (Pucino et al., 2019). Lactate transporter expression in the synovia correlated with T cell number in rheumatoid arthritis patients (Haas et al., 2015). Moreover, in contrast to healthy T cells, naive CD4+ T cells from rheumatoid arthritis patients were unable to upregulate the glycolytic enzyme PFKFB3, resulting in a state-of-energy deprivation and senescence (Pucino et al., 2019). These results suggest that lactate may contribute to T cell dysfunction in arthritis. More research should be done to understand how lactate levels contribute to disease progression in autoimmunity.

### Asthma and Allergic Disease

Systemic elevations in lactate have also been reported in asthma and allergic disease. Elevated plasma lactate has been measured in asthmatic patients, which correlated with reduced pulmonary function measured by forced expiratory volume in 1s (FEV1; Ostroukhova et al., 2012). These elevations were only ~1mM above normal serum levels; however, patients with stable asthma, rhinitis, and eczema also have lower lung pH as measured by exhaled breath condensate (EBC) than controls, and acute asthmatics have even lower EBC pH compared to the other groups (Brunetti et al., 2006). One potential explanation for the association between lactate and asthmatic severity is that patients with severe and steroid resistant asthma often have elevated Th2-driven inflammation and Th17-driven neutrophilic infiltration (Newcomb and Peebles, 2013; Irvin et al., 2014), both of which are augmented by lactate and the associated H^+^ ions.

However, lactic acid suppressed both IgE and IL-33-induced mast cell activation (Abebayehu et al., 2016, 2019), which should logically improve allergic disease. These effects occurred in culture, but also in a model of IL-33-induced peritonitis and IgE-driven passive anaphylaxis. Moreover, lactic acid-producing *lactobacilli* probiotic strains have been shown to improve asthma and allergic disease (Żukiewicz-Sobczak et al., 2014), although these results could be attributed to the tissue microbiome and additional immunomodulatory metabolites like butyrate. Thus, asthma is another example of cell-specific lactic acid effects that are poorly understood and need to be unraveled to move beyond disease association.

### Obesity

Patients with obesity and diabetes have higher plasma lactate concentrations than healthy volunteers (Chen et al., 1993); however, there is little known about the role of lactate in obesity. Adipocytes regularly produce lactate, which increases with adipocyte size, cell density (*ex vivo*), and epinephrine or insulin stimulation (Digirolamo et al., 1992; Krycer et al., 2020). Fat cells from obese or diabetic rats (or humans) can metabolize lactate to as much as 50–70% of imported glucose. Additionally, lactate affects cell redox, beta-oxidation, and lipolysis in adipocytes (Brooks, 2018). These complex effects again show that cell-specific lactate effects must be uncovered to understand and target pathways in disease.

Interestingly, while lactate production from lean adipocytes could logically support resident M2-like adipose tissue macrophages, obesity is associated with inflammatory macrophages that undergo glycolysis and also produce lactate (Caslin and Hasty, 2019). These macrophages have been shown to promote insulin resistance and promote diabetes (Lumeng et al., 2007). This contradiction between lactate production and inflammatory phenotype is poorly understood, and it remains plausible that metabolism influences their role in adipose tissue (O’Neill et al., 2016). Interestingly, these cells are similar to some tumor-associated macrophages (Helm et al., 2014; de-Brito et al., 2020); in that obese adipose tissue macrophages increased both M1- and M2-like markers and increased both glycolytic and oxidative metabolism, suggesting a mixed phenotype (Kratz et al., 2014; Boutens et al., 2018; Li et al., 2019). As lactate is elevated in both the tumor microenvironment and adipose tissue, future studies should explore the role of lactate in the unique polarization status of macrophages in these environments. Both tumor-associated macrophages and obese adipose macrophages have roles in lipid and iron handling (Kratz et al., 2014; Orr et al., 2014; Jung et al., 2017; Su et al., 2020), suggesting microenvironmental stimuli may modulate cell polarization and function. Like sepsis and asthma, we do not know the extent to which lactate is beneficial vs. pathological in obesity or how lactate acts in obesity-related diabetes.

The above should demonstrate the variety of diseases affected by elevated lactate levels, but we would like to point out that this is not a comprehensive list. Lactate therapy has been proposed for diseases like pancreatitis (Wu et al., 2011; Hoque et al., 2014) and myocardial infarction (Zhang et al., 2021). In many disease models, increasing buffering capacity or modulating lactate transporter expression may improve disease outcomes (Raghunand et al., 1999; Chirasani et al., 2013; Ohashi et al., 2013; Pilon-Thomas et al., 2016; Feichtinger and Lang, 2019; Pucino et al., 2019). Many studies have also attempted to administer probiotic lactobacilli strains, lactate producing polymers, or lactate infusions (Herías et al., 2005; Wu et al., 2011; Żukiewicz-Sobczak et al., 2014; Iraporda et al., 2016). Together, these emphasize that understanding the effects of lactate on inflammation may be critical and fruitful for many disease states.

## Conclusion

Immunologists are just now beginning to understand the role of lactate and the associated H^+^ ions as metabolites, feedback regulators, and signaling molecules within the immune system and more broadly within physiology. Lactate and H^+^ ions generally act to suppress glycolytic ATP production, contributing to reduced inflammatory cell signaling and mediator production. However, lactate with and without the associated H^+^ ions also have specific receptor-mediated functions and can promote Th2 and Th17 immunity. Additionally, lactate and the associated H^+^ ions appear to promote a mixed M1/M2 phenotype in tumor-associated macrophages, suggesting a potential role for additional signaling mediators in the microenvironment.

The implications for some of the seemingly contradictory effects of lactate with and without the associated H^+^ ions are not clear. Studies suggest that Th2 and Th17 responses may coordinate in anti-helminth immune defense, suggesting an evolutionary role for promoting both responses. However, the suppressive effects of lactate and the associated H^+^ ions on mast cell activation complicate this hypothesis. Moreover, lactate is elevated in many disease conditions and appears to have both detrimental and beneficial effects. Following acute immune cell activation, such as in wound healing, lactic acid may act as a negative feedback regulator. In chronic inflammation, elevated lactate levels may represent an inability to control inflammation or respond to feedback.

While many murine studies have attempted to modulate lactate and the associated H^+^ ions, there is still much to unravel about the contribution of cell, receptor, mediator, and microenvironment effects. It would be useful for researchers to further investigate the specific role of lactate vs. H^+^ in each immune cell subset, as well as the contribution of intracellular vs. extracellular (paracrine/endocrine) lactate production. Additionally, it would be useful to further clarify the contribution of lactate production and clearance to each disease and the role of lactate in both immune and non-immune cell types. If lactate oxidation is beneficial for one cell type yet detrimental for another, targeting specific cell types or specific signaling mechanisms will be important. Finally, future studies should further explore the mechanisms by which lactate and the associated H^+^ ions suppress Th1-mediated inflammation while promoting M2- and Th17-driven responses, which may help to identify and develop more effective therapeutic targets for diseases like cancer, sepsis, allergic diseases, and autoimmune diseases. Thus, while we still have much to learn, it is evident that lactate and the associated H^+^ ions have systemic influence on not only the immune system, but physiology in health and disease.

## Author Contributions

HC was invited to write the review and wrote the initial manuscript draft, while all authors contributed to idea development and manuscript edits. Moreover, HC, DA, and JR contributed to previous publications from the Ryan Lab which sparked our interest and understanding of this topic. All authors contributed to the article and approved the submitted version.

## Funding

HC is currently funded by an AHA postdoctoral fellowship (20POST35120547), DA is currently funded by a NIH postdoctoral fellowship (5F32HL147405-02), JP is currently funded by a NIH institutional training grant (5T32HL144446-02), and JR is funded by NIH research grant (1R01AI138495).

## Conflict of Interest

The authors declare that the research was conducted in the absence of any commercial or financial relationships that could be construed as a potential conflict of interest.

## Publisher’s Note

All claims expressed in this article are solely those of the authors and do not necessarily represent those of their affiliated organizations, or those of the publisher, the editors and the reviewers. Any product that may be evaluated in this article, or claim that may be made by its manufacturer, is not guaranteed or endorsed by the publisher.

## Supplementary Material

Supplementary Table 1 Detailed findings reported in lactate-immune literature. Effect on inflammation noted by color: blue=decreases, orange=increases, green=both. Cell type abbreviations: PBMC, peripheral blood mononuclear cell; (i) BMDM, (immortalized) bone marrow-derived macrophage; MDDC, monocyte-derived dendritic cell; MDM, monocyte-derived macrophage; MDSC, monocyte-derived suppressor cells; BMMC, bone marrow-derived mast cells; BMDM, bone marrow-derived macrophage; BMMC, bone marrow-derived mast cells; BMDC, bone marrow-derived dendritic cells; NK, natural killer; CTL, CD8^+^ or cytotoxic killer T cell. Treatment: H_3_PO_4_, phosphoric acid; HCl, hydrochloric acid; GM-CSF, granulocyte-macrophage colony-stimulating factor; NaOH, sodium hydroxide; TCM, tumor-conditioned media. Activation signal abbreviations: BCG-CWS, Bacille Calmette-Guerin cell wall skeleton; LPS, lipoteichoic acid; PGN, peptidoglycan; LTA, lipoteichoic acid; OVA, ovalbumin; Pam3Cys, Pam3Cys-Ser-(Lys)4; Poly(I:C), polyinosinic/polycytidylic acid; PMA, phorbol myristate acetate. Effector molecules: AKT, protein kinase B; AP-1, activator protein 1; ARG1, arginase 1; ATP6V0d2, macrophage-specific V-ATPase subunit; BTK, Bruton’s tyrosine kinase; CCl, chemokine ligand (C-C motif); CD, cluster of differentiation; CSF, colony stimulating factor; COX2, cyclooxygenase-2; Cxcl, chemokine ligand (C-X-C Motif); ERK, extracellular signal-regulated kinase; GEM, GTP binding protein overexpressed in skeletal muscle; GPR, G-protein coupled receptor; HIF, hypoxia inducible factor; HK, hexokinase; ICER, inducible cyclic AMP early repressor; IFN, interferon; Ig, immunoglobulin; IL-, interleukin; JNK, c-Jun N-terminal kinase; LIF, leukemia inhibitory factor; M, macrophage; MAVS, mitochondrial antiviral signaling protein; MCP, monocyte chemoattractant protein; MCT, monocarboxylic transporter; MD2, myeloid differentiation factor 2; MIP, macrophage inflammatory protein; miR, microRNA; MMP, matrix metalloprotease; mTORC, mammalian target of rapamycin complex; NFκB, nuclear factor kappa B; p, protein kinase; PGE2, prostaglandin E2; PEPCK, phosphoenolpyruvate carboxykinase; PTX3, pentraxin 3; RIG1, retinoic acid-inducible gene 1; RORγt, retinoic acid receptor-related orphan receptor gamma; ROS, reactive oxygen species; STAT, signal transducer and activator of transcription; SYK, spleen tyrosine kinase; TAK1, transforming growth factor-β-activated kinase 1; TCR, T cell receptor; TNF, tumor necrosis factor; VEGF, vascular endothelial growth factor.
